# Physicians and pharmacists’ clinical knowledge of statin therapy and monitoring parameters, and the barriers to guideline implementation in clinical practice

**DOI:** 10.1371/journal.pone.0280432

**Published:** 2023-01-20

**Authors:** Fahmi Y. Al-Ashwal, Syed Azhar Syed Sulaiman, Siti Maisharah Sheikh Ghadzi, Mohammed Abdullah Kubas, Abdulsalam Halboup

**Affiliations:** 1 Discipline of Clinical Pharmacy, School of Pharmaceutical Sciences, Universiti Sains Malaysia, Penang, Malaysia; 2 Department of Clinical Pharmacy and Pharmacy Practice, Faculty of Pharmacy, University of Science and Technology, Sana’a, Yemen; 3 Clinical Pharmacy Department, University of Science and Technology Hospital (USTH), Sana’a, Yemen; 4 School of Pharmacy & Medical Sciences, Lebanese International University (LIU), Sana’a, Yemen; University of South Australia, AUSTRALIA

## Abstract

**Background:**

Millions of individuals worldwide use statins, and their significant impact on cardiovascular disease (CVD) has been well-established. However, a lack of knowledge about the up-to-date guideline recommendations regarding statin therapy is a common barrier to implementation in clinical practice. Therefore, the present study aimed to assess the current clinical knowledge about statin therapy and its monitoring parameters. Also, we evaluated the barriers to cholesterol management guideline implementation in Yemen.

**Methods:**

This observational cross-sectional study was conducted over four months, from June/2021 to September/2021, in Sana’a, Yemen. A validated questionnaire was distributed face-to-face to 650 participants (350 physicians and 300 pharmacists). Physicians and pharmacists from governmental and private hospitals and those working in private clinics or community pharmacies were included in the study.

**Results:**

A total of 496 participants filled out the survey, with 22 being excluded due to incomplete data. So, the study has an overall response rate of 72.9% (474). The majority of pharmacists (81.8%) and physicians (78.7%) could not identify the patient group that needed ASCVD risk assessment before statin therapy initiation. Although a significant proportion of respondents knew of the fact that high-intensity statins are recommended for patients with ASCVD (65.4%) and primary hypercholesterolemia (58.4%), the majority of physicians and pharmacists could not identify the high (61.6% and 66.7.3%, respectively) and moderate statin-intensity doses (72.2% and 68.6%, respectively). Only 21.9% of all respondents knew that atorvastatin and rosuvastatin can be administered at any time of the day. Similarly, a low overall rate of respondents (19.6%) knew that atorvastatin does not need dose adjustment in chronic kidney diseases, with a statistically significant difference in knowledge between physicians and pharmacists (12.5% vs. 25.6%, *p* <0.001, respectively). Notably, only 39.2% of participants were aware that statins are not safe to use during breastfeeding. Around half of respondents (52.3%) correctly identify the duration (4 to 12 weeks) at which LD-C measuring is recommended after therapy initiation or dose change. The lowest knowledge scores for respondents were related to statin-drug interactions. Age, experience, degree, and previous guideline exposure were all significantly associated with the knowledge scores (p <0.05). The four most perceived barriers to implementing cholesterol management guidelines were no audit on adherence to the guidelines in the workplace (73.4%), insufficient resources to adequately implement and follow up on the guideline’s recommendations (73.6%), patient’s financial status (75.7%), and lack of familiarity about the guideline’s latest recommendations (63.3%).

**Conclusion:**

Physicians and pharmacists had suboptimal clinical knowledge regarding statin therapy, dose intensities, drug-drug interaction, contraindications, and monitoring parameters. Therefore, physicians’ and pharmacists’ educational interventions regarding the up-to-date recommendation about statins are recommended.

## Introduction

Millions of individuals worldwide use statins, and their significant impact on cardiovascular disease (CVD) has been well-established. For instance, numerous clinical trials and meta-analysis studies have demonstrated the significant benefits of statins in reducing major cardiovascular events [1–4]. As a result, the clinical practice guidelines recommend statins in primary and secondary CVD prevention [5–7]. For example, the latest 2018 ACC/AHA multi-society guideline on the management of blood cholesterol provides strong recommendations for initiating statin therapy for patients with ASCVD, DM (40–75 years), and primary hypercholesterolemia (≥190mg /dl). These three groups of patients do not need a risk assessment to initiate statin therapy. The fourth statin-benefit group is individuals aged 40–75 years old, but a 10-year ASCVD risk assessment should be performed for this age category [8].

However, healthcare providers’ lack of knowledge about up-to-date guideline recommendations is one of the most common barriers to guideline implementation in clinical practice [9]. Previous studies have reported knowledge gaps among physicians and/or pharmacists regarding cholesterol management guidelines and/or CVD risk assessment before statin therapy initiation [10–13]. For example, two studies from the USA that were done among healthcare providers [14] and clinical pharmacists [15] reported several knowledge gaps about guidelines, particularly those related to ASCVD risk assessment and statin therapy clinical indications. In Singapore, only 35% of clinicians were familiar with the 2013 ACC/ AHA guidelines [16]. Notably, these studies were not specific to all clinical aspects of statin therapy (clinical indications, dose intensities, statin-drug interactions, contraindications, and monitoring parameters). Therefore, evaluating the knowledge regarding these aspects for statins is of paramount for safe and effective use in practice.

In Yemen, no previous study has evaluated physicians’ and pharmacists’ clinical knowledge of statin therapy. Moreover, no data is available about the barriers to guideline implementation in clinical practice. Therefore, the present study aimed to assess the current clinical knowledge about statin therapy and its monitoring parameters. Also, we evaluated the barriers to cholesterol management guideline implementation in Yemen. By identifying the knowledge gaps and barriers to guideline implementation, appropriate interventions could be designed and implemented for the appropriate utilization of statin therapy in clinical practice.

## Methods

### Ethical approval

The Ethical Committee of the Medical Research, University of Sciences and Technology, Sana’a, Yemen (EAC/UST193) approved this project (this study is part of a project about statin therapy in Yemen). The ethical committee approved verbal informed consent as the participants’ identities are entirely anonymous, and the study does not involve any risk. Therefore, healthcare professionals who gave their verbal consent and agreed to participate were included in the study. All participants received adequate explanations of the study’s aims and were given the opportunity to ask questions.

### Study design and setting

This observational cross-sectional study was conducted based on a validated self-administered questionnaire (S1 File). The study was conducted over four months, from June/2021 to September/2021, in Sana’a, Yemen. The questionnaires were administered face-to-face to participants in five major tertiary governmental hospitals, 12 private hospitals, and more than 100 community pharmacies. Also, private clinics were targeted so that we get a representative sample of physicians at different workplaces. A convenience sampling approach was used to select the participants.

The study included physicians (consultants, specialists, resident doctors, and general practitioners) working in private and governmental hospitals and private clinics. Physicians with the following specialties were targeted: internal medicine, cardiology, chronic kidney disease, endocrinology, and general prescribers. Also, the study included licensed pharmacists working in private and governmental hospitals and community pharmacies with different degrees (PharmD, B.Pharm, and diploma). Exclusion criteria: Physicians from the psychiatric, surgery, and intensive care departments. Also, physicians who do not want to participate in the study. Moreover, physicians and pharmacists who were in training or new graduates (<6 months).

### Sample size calculation and participants

The total number of pharmacists in 2019 was reported to be approximately 18000 [17]. Also, according to the 2014 annual health statistics report, Sana’a has a total of 1732 physicians [18]. Therefore, with a 95% confidence interval and 5% precision, and based on the assumption that 50% of physicians and pharmacists will have optimal knowledge regarding statin therapy prescription for patients with clinical indications, the calculated sample size will be 385, adding a 10% non-response rate, the total number will be approximately 428. This calculation was based on the Daniel sample size formula [19]. We have distributed approximately 650 questionnaires (350 among the physician and 300 among pharmacists).

### Study instrument and scoring system

The questionnaire was developed based on the guidelines’ recommendations, previous literature, and experts’ opinion [5, 20]. For the barriers to guideline implementation section, the choices were adapted from previously published research [20].

The questionnaire consists of 4 sections (S1 File). Participants’ age, gender, qualifications, workplace, and experience were all included in the first section. Additionally, this section included a question about whether participants had read any dyslipidemia guidelines in the previous three years. The second section included fifteen questions designed to assess the participants’ overall clinical knowledge of statin therapy. This section will examine participants’ knowledge of clinical indications for statin therapy, their awareness of the terms high, moderate, and low dose intensity, and their knowledge of the appropriate dose intensity for patients with high ASCVD. Additionally, because some statins can be administered at any time of day, we want to assess whether participants were aware of them. Another question was about statin medication selection in CKD patients. Following that are three questions regarding statin-drug interactions and three more for statin contraindications. Section three featured three questions designed to measure participants’ understanding of monitoring parameters recommended for the safety and efficacy of statin therapy. The final section investigated physicians’ and pharmacists’ perceptions of impediments to properly implementing cholesterol management guidelines in Yemeni clinical practice.

A total of fifteen questions were utilized to assess clinical knowledge about statin therapy, and three questions were for the monitoring parameters knowledge section. The correct answer received one point, while the incorrect or I don’t know answers were given zero points. Therefore, for the total 15 questions regarding statin therapy clinical knowledge, the total scores ranged from 0 to 15.

### Validation

A panel of experts representing both the pharmacy and medical departments validated the questionnaires’ content for content validity. Among these experts are one clinical pharmacist, two clinical pharmacy lecturers, a lecturer from the medical faculty, and two consultants from the internal medicine and cardiology departments. They were requested to assess the content relevance, appropriateness, and representation of the evaluated tool. Modifications were made in response to the experts’ feedback, and the scale-content validity index based on the universal agreement (S-CVI/UA) was calculated. A value of 0.8 or higher was used to reflect a satisfactory level of content validity for the domains [21]. The S-CVI/UA for clinical knowledge of statin therapy, monitoring parameters, and barriers were 0.80, 1, and 0.92, respectively.

For face validity, the questionnaires were given to several individuals from the target population that will respond later to the questionnaire. The participants were asked to provide feedback on the construct’s clarity and grammatical and typographical errors. Most importantly, to report any ambiguous questions. The main goal of face validity is to modify the question structure and make the questionnaire more appealing and understandable to the participants. For reliability, the questionnaire was piloted-tested on 40 participants. Cronbach’s alpha ≥0.7 was regarded as acceptable [22]. The reliability analysis was done on the subscales as the questionnaire consisted of different factors (1. Clinical indications and dose intensities (9 items), 2. Statin-drug interaction (3 items), 3. Contraindications (3 items), 4. Knowledge of monitoring parameters (3items)). The Cronbach’s alpha ranged between 0.703 and 0.713, indicating that the questionnaire’ subscales had acceptable internal consistency.

### Statistical analysis

The data were analyzed using SPSS, version 25.0 (IBM Corp., Armonk, NY, USA). We assessed the normality using the Kolmogorov–Smirnov test (number ≥50). The data were not normally distributed. The Kolmogorov–Smirnov was statistically significant (p <0.05), indicating non-normal distribution. Therefore, we used the Kruskal-Wallis test and Mann-Whitney U test to assess the association between knowledge scores and participants’ characteristics. The shape of scores distribution for the groups of an independent variable (e.g., the shape of female scores vs. male scores) was not similar. Therefore, the mean rank score for these tests was utilized to express the scores instead of the median [23]. The higher the mean rank score (or median), the higher the knowledge level. Also, a Chi-square test was employed to test for association between categorical variables.

## Results

### Participants characteristics

 Out of the 650 healthcare providers (300 pharmacists and 350 physicians) who were approached to participate in the study, 496 filled out the survey, with 22 being excluded due to incomplete questionnaires. So, the study has an overall response rate of 72.9% (474). A total of 258 pharmacists (54.4%) and 216 physicians (45.6%).

Table 1 summarizes the participants’ demographic characteristics. Overall, the majority of respondents were males (69.6%), aged > = 30 years old (62%), and 57.2% of them had six or more years of experience (57.2%). The majority of pharmacists had a BSc degree (60.5%) and were working in community pharmacies (73.6%). On the other hand, the majority of physicians were working in private and governmental hospitals (82%), with only 18% in private clinics. Over two-fifths of physicians (46.3%) and a minority of pharmacists (15.9%) reported reading a guideline on cholesterol management during the last three years.

**Table 1:** Demographic characteristics (n = 474).

Category	Sub-category	Physicians F (%) (n = 216)	Pharmacists F (%) (n = 258)	Total F (%) (n = 474)
Gender	Male	147 (68.1)	183 (70.9)	330 (69.6)
	Female	69 (31.9)	75 (29.1)	144 (30.4)
Age	< 30	71 (32.9)	109 (42.2)	180 (38)
	≥ 30	145 (67.1)	149 (57.8)	294 (62)
Current workplace	Private hospital	98 (45.4)	42 (16.3)	140 (29.5)
	Governmental hospital	79 (36.6)	26 (10.1)	105 (22.2)
	Private clinic	39 (18)	-	39 (8.2)
	Community pharmacy	-	190 (73.6)	190 (40.1)
Degree	Consultant	29 (13.4)	-	29 (6.1)
	Specialist	44 (20.4)	-	44 (9.3)
	Resident	35 (16.2)	-	35 (7.4)
	GP	108 (50)	-	108 (22.8)
	PharmD	-	26 (10)	26 (5.5)
	B Pharm	-	156 (60.5)	156 (32.9)
	Diploma	-	76 (29.5)	76 (16)
Experience years	<6	108 (50)	95 (36.8)	203 (42.8)
	≥6	108 (50)	163 (63.2)	271 (57.2)
Read guidelines on cholesterol management in the past 3 years	Yes	100 (46.3)	41 (15.9)	141 (29.7)
	No	116 (53.7)	217 (84.1)	333 (70.3)

### Clinical knowledge about statin therapy

Table 2 shows the clinical knowledge of statin therapy among pharmacists and physicians. Most pharmacists (81.8%) and physicians (78.7%) could not identify the patient group that needed ASCVD risk assessment before statin therapy initiation. Less than a third of surveyed physicians and pharmacists (31.5% and 29.1%, respectively) were aware of high, moderate, and low-intensity statins definitions. Although a significant proportion of respondents knew of the fact that high-intensity statins are recommended for individuals with ASCVD (65.4%) and primary hypercholesterolemia (58.4%), the majority of them could not identify the high (61.6% and 66.7.3%, respectively) and moderate statin doses (72.2% and 68.6%, respectively). Similarly, only two-fifths of physicians and pharmacists were aware of the recommended dose intensity for the DM patient case (40.7%).

**Table 2:** Participants’ clinical knowledge about statin therapy.

Question	Physicians F (%) (n = 216)	Pharmacists F (%) (n = 258)	Total F (%) (n = 474)	P-value*
**It is recommended to initiate statin therapy without ASCVD risk assessment in all the following patients EXCEPT (Obese and a smoker patient aged 42 years old)**				
Correct	46 (21.3)	47 (18.2)	93 (19.6)	0.400
Incorrect	170 (78.7)	211 (81.8)	381 (80.4)	
**What percent of LDL-C reduction would you expect from low, moderate, and high-intensity statin daily therapy? (< 30% for low, 30- <50% for moderate, and ≥50% for high-intensity statin therapy)?**				
Correct	68 (31.5)	75 (29.1)	143 (30.2)	0.569
Incorrect	148 (68.5)	183 (70.9)	331 (69.8)	
**Which of the following daily doses of statin therapy is considered a high-intensity statin? (Rosuvastatin 20mg)**				
Correct	83 (38.4)	86 (33.3)	169 (35.7)	0.249
Incorrect	133 (61.6)	172 (66.7)	305 (64.3)	
**Which of the following daily doses of statin therapy is considered a moderate-intensity statin: (Rosuvastatin 10 mg)**				
Correct	60 (27.8)	81 (31.4)	141 (29.7)	0.391
Incorrect	156 (72.2)	177 (68.6)	333 (70.3)	
**Which of the following statin intensity is recommended for adult patients with clinical ASCVD such as myocardial infarction and angina: (High-intensity statin)**				
Correct	146 (67.6)	164 (63.6)	310 (65.4)	0.359
Incorrect	70 (32.4)	94 (36.4)	164 (34.6)	
**Which of the following statin intensity is recommended for patients with severe primary hypercholesterolemia (LDL-C level ≥190 mg/dL [≥4.9 mmol/L]): (High-intensity statin)**				
Correct	143 (66.2)	134 (51.9)	277 (58.4)	0.002
Incorrect	73 (33.8)	124 (48.1)	197 (41.6)	
**Which of the following statin-intensity is recommended for patients with only diabetes mellitus aged 40–75 years old with LDL-C level of 70 mg/dL or more: (Moderate-intensity statin)**				
Correct	87 (40.3)	106 (41.1)	193 (40.7)	0.859
Incorrect	129 (59.7)	152 (58.9)	281 (59.3)	
**Which of the following statin medications can be taken by patients at any time of the day? (Atorvastatin and Rosuvastatin)**				
Correct	28 (13)	76 (29.5)	104 (21.9)	<0.001
Incorrect	188 (87)	182 (70.5)	370 (78.1)	
**Which of the following statin medications does not need dose adjustment in chronic kidney disease: (Atorvastatin)**				
Correct	27 (12.5)	66 (25.6)	93 (19.6)	<0.001
Incorrect	189 (87.5)	192 (74.4)	381 (80.4)	
**Which of the following statin medications is associated with clinically significant drug-drug interaction when used in combination with amlodipine: (Simvastatin)**				
Correct	29 (13.4)	59 (22.9)	88 (18.6)	0.008
Incorrect	187 (86.6)	199 (77.1)	386 (81.4)	
**Which of the following statin medications is preferred to use for patients on warfarin to avoid drug-drug interactions: (Atorvastatin)**				
Correct	40 (18.5)	64 (24.8)	104 (21.9)	0.099
Incorrect	176 (81.5)	194 (75.2)	370 (78.1)	
**Which of the following statin medications is the safest to use in patients taking clarithromycin: (Rosuvastatin)**				
Correct	39 (18.1)	68 (26.4)	107 (22.6)	0.031
Incorrect	177 (81.9)	190 (736)	367 (77.4)	
**Statin therapy is contraindicated in pregnancy: (Yes)**				
Correct	126 (58.3)	154 (59.7)	280 (59.1)	0.765
Incorrect	90 (41.7)	104 (40.3)	194 (40.9)	
**Statin therapy can be used safely during breastfeeding: (No)**				
Correct	81 (37.5)	105 (40.7)	186 (39.2)	0.478
Incorrect	135 (62.5)	153 (59.3)	288 (60.8)	
**Statin therapy is contraindicated active liver disease: (Yes)**				
Correct	169 (78.2)	162 (62.8)	331 (69.8)	<0.001
Incorrect	47 (21.8)	96 (37.2)	143 (30.2)	

*Significant difference between Pharm-D and medical students using Chi-square test. ASCVD (Atherosclerotic Cardiovascular disease), Total F (Total frequency), LDL-C (Low-density lipoprotein-cholesterol)

For the knowledge regarding the intake time of statins, most participants have the misinformation that all statins must be administered at night, with only 21.9% of all respondents knowing that atorvastatin and rosuvastatin can be administered at any time of the day. The Chi-Square test revealed that a lower percentage of physicians were aware of this information than pharmacists (13% vs. 29.5%, *p* <0.001). Similarly, a low overall rate of respondents (19.6%) knew that atorvastatin does not need dose adjustment in chronic kidney diseases, with a statistically significant difference in knowledge between physicians and pharmacists (12.5% vs. 25.6%, *p* <0.001, respectively).

The lowest knowledge scores for respondents were related to SDIs. In this light, only 18.6% of respondents correctly identified the clinically significant SDIs between simvastatin and amlodipine. Similarly, only 21.9% of participants knew that atorvastatin has the safest DDI profile when used with warfarin. In addition, just over a fifth of respondents (22.6%) knew that rosuvastatin is the safest statin (of the given choices) to use in patients taking clarithromycin. Notably, physicians had lower knowledge regarding amlodipine-simvastatin DDI than pharmacists (13.4 vs. 22.9%, *p* = 0.008). Likewise, there was a significant difference between physicians and pharmacists regarding the safest statin agent to use with clarithromycin (18.1% vs. 26.4%, *p* = 0.031). Regarding statins contraindications, a large proportion of respondents were aware that statins are contraindicated in pregnancy (59.1%) and active liver disease (69.8%). Physicians had a higher knowledge regarding the contraindication of statins in active liver disease compared to pharmacists (78.2% vs. 62.8%, *p* <0.001). Notably, a lower percentage of participants (39.2%) were aware that statins are not safe to use during breastfeeding.

The association between sociodemographic and statin clinical knowledge is presented in Table 3. There were significant associations between respondents’ mean rank clinical knowledge of statins and participants’ characteristics except for the gender and profession groups. Patients younger than 30 years old and with <6 experience years had significantly lower knowledge scores compared to those older than 30 years old and with ≥6 years of experience (*p* <0.001).

**Table 3:** Factors influencing clinical knowledge of statin therapy.

Variable	Frequency (%)	Mean rank	Test value	P-value^a^
**Gender** ^a^				
Male	330	232.17	25520	0.196
Female	144	249.72		
**Age** ^a^				
< 30	180	196.02	33927	<0.001
≥ 30	294	262.90		
**Profession** ^a^				
Physician	216	235.41	28135	0.760
Pharmacist	258	239.25		
**Workplace** ^b^				
Private hospital	140	264.75	21.339	<0.001
Governmental hospital	105	265.16		
Private clinic	39	224.45		
Community pharmacy	190	204.81		
**Experience Years** ^a^				
<6	203	201.73	34767	<0.001
≥6	271	264.29		
**Read a guideline on cholesterol management** ^a^				
Yes	141	346.39	38830	<0.001
No	333	191.39		
**Physicians’ degree** ^b^ ^,^ ^c^				
Consultant	29	156.5	56.454	<0.001
Specialist	44	144.2		
Resident	35	114.23		
GP	108	79.21		
Consultant vs. Specialist				1.000
Consultant vs. Resident				0.040
Consultant vs. GP				<0.001
Specialist vs. Resident				0.196
Specialist vs. GP				<0.001
Resident vs. GP				0.022
**Pharmacists’ degree** ^b^ ^,^ ^c^				
PharmD	26	201.83	59.801	<0.001
B Pharm	156	140.64		
Diploma	76	81.89		
PharmD vs. B Pharm				<0.001
PharmD vs. Diploma				<0.001
B Pharm vs. Diploma				<0.001

^a^Mann-Whitney U test,

^b^Kruskal-Wallis test,

^c^ Dunn’s post hoc tests are carried out on each pair of the groups.

GP (General practitioner), B Pharm (Bachelor of Pharmacy)

The Mann-Whitney U and the Kruskal-Wallis tests (Table 3) showed a significant difference (χ2 = 56.454, p < 0.001) among the physicians’ degree groups with a mean rank knowledge score of 156.5 for consultants, 144.2 for specialists, 114.23 for resident, and 79.21 for GPs. Similarly, there was a statistically significant difference (χ2 = 59.801, p < 0.001) among the pharmacists’ degree categories with a mean rank knowledge score of 201.83, 140.64, and 81.89 for PharmD, bachelor, and diploma holders, respectively.

### Knowledge of monitoring parameters

The vast majority of the surveyed pharmacists (92.2%) and physicians (95.8%) were aware that lipid profile is recommended at baseline before initiating statin therapy (Table 4). More than two-fifths of the physicians (42.1%) and pharmacists (44.6%) thought HbA1c or fasting glucose testing was recommended before statin therapy. For checking liver enzymes (ALT), a significantly higher percentage of physicians than pharmacists chose it as a recommended test before statin initiation (77.3% vs. 30.2%, p <0.001). For efficacy monitoring of statins, around half of respondents (52.3%) correctly identify the duration (4 to 12 weeks) at which LD-C measuring is recommended after therapy initiation or dose change. On the other hand, the majority of participants (87.8%) were aware that a follow-up lipid profile is recommended every 3–12 months as clinically needed. For monitoring parameters, a significantly higher percentage of physicians knew when to do a lipid profile for efficacy monitoring after statin therapy initiation (p = 0.042 and 0.001).

**Table 4:** Participants’ knowledge of monitoring parameters.

Question	Physicians F (%) (n = 216)	Pharmacists F (%) (n = 258)	Total F* (%) (n = 474)	P-value*
**What is/are the lab tests recommended for patients at baseline before initiating statin therapy: (please choose all that apply)**				
Lipid profile	207 (95.8)	238 (92.2)	445 (93.9)	0.105
HbA1c or fasting glucose	91 (42.1)	115 (44.6)	206 (43.5)	0.593
Liver enzymes (ALT)	167 (77.3)	78 (30.2)	245 (51.7)	<0.001
Creatinine kinase (CK)	44 (20.4)	46 (17.8)	90 (19)	0.482
**It is recommended to assess statin efficacy by measuring LDL-C ……………… after statin therapy initiation or dose change: (4 to 12 weeks)**				
Correct	124 (57.4)	124 (48.1)	248 (52.3)	0.042
Incorrect	92 (42.6)	134 (51.9)	226 (47.7)	
**Once the patient achieved the target lipid level, it is recommended to do a lipid profile every: (3–12 months as needed)**				
Correct	201 (93.1)	215 (83.3)	416 (87.8)	0.001
Incorrect	15 (6.9)	43 (16.7)	58 (12.2)	

*Total F (Total frequency)

### Barriers to guideline implementation

The four most perceived barriers to implementing cholesterol management guidelines (Table 5) were no audit on adherence to the guidelines in the workplace (73.4%), insufficient resources to adequately implement and follow up on the guideline’s recommendations (73.6%), patient’s financial status (75.7%), and lack of familiarity about the guideline’s latest recommendations (63.3%). The lowest perceived barriers were those related to the guideline, such as being too long (5.9%), changing too often (6.8%), and its complexity and difficulty of use (11.6%). Moreover, a minority of respondents (14.3%) disagree with the guideline recommendations.

**Table 5:** Participants reported barriers to proper implementation of guideline recommendations regarding statin therapy.

Barriers	Physicians F (%) (n = 216)	Pharmacists F (%) (n = 258)	Total F (%) (n = 474)	P-value
1. Not very familiar with the guideline latest recommendations for statin therapy	125 (57.9)	175 (67.8)	300 (63.3)	0.025
2. Workload and lack of time prevent the application of guideline recommendations regarding statin medication	159 (73.6)	105 (40.7)	264 (55.7)	<0.001
3. Patients’ financial status does not allow them to implement the guideline recommendation	160 (74.1)	199 (77.1)	359 (75.7)	0.439
4. Adherence to the international guidelines will not make a difference in the clinical outcomes of Yemeni patients	68 (31.5)	119 (46.1)	187 (39.5)	0.001
5. Disagreement with some of the guideline recommendations regarding statin therapy	32 (14.8)	36 (14)	68 (14.3)	0.790
6. Guidelines’ recommendations are complex and difficult to use	18 (8.3)	37 (14.3)	55 (11.6)	0.042
7. The cholesterol management guidelines are too long	22 (10.2)	6 (2.3)	28 (5.9)	<0.001
8. The cholesterol management guideline changes too often	24 (11.1)	8 (3.1)	32 (6.8)	0.001
9. The cholesterol management guideline is too rigid to apply to all individual patients	47 (21.8)	54 (20.9)	101 (21.3)	0.826
10. No follow-up or audit on adherence to the guidelines in the workplace	146 (67.6)	202 (78.3)	348 (73.4)	0.009
11. Insufficient resources to adequately implement and follow up the guideline’s recommendations	157 (72.7)	192 (74.4)	349 (73.6)	0.670
12. Patient’s noncompliance with statins due to side effects (e.g. myalgia)	43 (19.9)	55 (21.3)	98 (20.7)	0.706

Regarding the differences between physicians and pharmacists regarding the perceived barriers to guideline implementation, physicians were more likely to perceive workload and lack of time as barriers compared to pharmacists (73.6% vs. 40.7%, p<0.001). Furthermore, more physicians than pharmacists believed that the cholesterol management guidelines were too long or changed too often (p <0.001 and p = 0.001, respectively). On the other hand, a significantly higher percentage of pharmacists than physicians perceived the complexity and difficulty of guideline use as a barrier (8.3% vs. 14.3%, p = 0.042). Similarly, lack of awareness about the guideline, lack of audit on adherence to the guidelines in the workplace, and the belief that adherence to the international guidelines will not make a difference in clinical outcomes of Yemeni patients were reported by more pharmacists than physicians (Table 5).

## Discussion

### Clinical knowledge about statin therapy

In Yemen, physicians and pharmacists had insufficient clinical knowledge about statin therapy and the up-to-date recommendations of the latest 2018 ACC/AHA guidelines for dyslipidemia management. Eleven out of 15 questions about clinical knowledge of statin therapy had less than 50% correct response rate. Previous studies reported a low awareness level of cholesterol guidelines among healthcare providers, particularly physicians and pharmacists. For example, physicians and clinical pharmacists in Suadi Arabia had insufficient awareness of the practice-changing recommendations of the 2013 ACC/AHA dyslipidemia management guidelines [10]. Moreover, just over a third of Singapore’s physicians (35%) were familiar with the 2013 ACC/AHA guidelines [16]. Furthermore, in a USA study, less than half of healthcare providers had read the guideline [14]. These findings are consistent with those of Reiner et al., 2010, who found that only half of the physicians utilize dyslipidemia management guidelines and that their knowledge was suboptimal [24]. Similarly, Heidrich et al. showed that only 63% of internists and 32% of GPs commenced lipid-lowering therapy in accordance with guidelines [25].

On the other hand, other studies reported overall good knowledge among healthcare providers regarding statin therapy and/or dyslipidemia management guidelines. For example, the general awareness and the use of the national lipid management guideline were good among postgraduate primary care trainees in Malaysia [26]. Similar findings were achieved by Hammad et al., who reported adequate knowledge and positive attitude among physicians and pharmacists regarding statin therapy utilization for type 2 diabetic patients [27]. In addition, a USA-based study found that the vast majority of clinical pharmacists (92%) had read the guideline, and 72% of participants were able to identify the four benefit groups for statin therapy [15]. Nevertheless, the same study reported knowledge gaps regarding lipids monitoring after statin initiation, and the knowledge gaps were higher in clinical pharmacists who practiced for more than ten years and specialized in internal medicine [15]. According to a survey conducted in Kuwait, over 90% of physicians reported using lipid guidelines and considered themselves knowledgeable about them. In the same study, younger physicians were found to have a lower level of knowledge and employed guidelines less frequently [28].

According to the 2018 AHA/ACC guidelines, patients with ASCVD, primary hypercholesterolemia, and DM aged 40–75 years old should be initiated statin therapy without ASCVD risk assessment. In the present study, most physicians and pharmacists (80.4%) could not identify the patient group that needs ASCVD risk assessment before initiating statin therapy. This knowledge gap among healthcare providers could result in the underutilization of statins among patients with clinical indications. Indeed, we recently have reported knowledge and practice gaps regarding CVD risk assessment among physicians and pharmacists [12, 13].

Another knowledge gap was related to the definition of high, moderate, and low-intensity statins, with more than two-thirds of respondents (69.8%) unaware of it. These observations were also in line with findings from the USA, with two-thirds of cardiology/endocrinology providers unaware of the definition of statin therapy intensity [14]. In contrast, a high percentage of clinical pharmacists in the USA (63.1%) were aware of the definitions of statin intensities. The lack of proper outcome anticipation among healthcare providers regarding different statin intensities could lead to inappropriate prescribing of statin therapy for patients with high CVD risk. In this light, most of the recent dyslipidemia guidelines recommend a reduction in LDL-C by 50% or more for secondary prevention or very high-risk patients, which is achieved by using high-intensity daily dose statin. Lack of knowledge about this information could indirectly reflect that the majority of physicians in Yemen do not use the % reduction in LDL-c to monitor the efficacy of statins, particularly among high-risk patients.

Notably, a significant proportion of respondents knew that high-intensity statins are recommended for patients with ASCVD (65.4%) and primary hypercholesterolemia (58.4%). However, the majority of them could not identify the high (64.3%) and moderate statin intensities (70.3%). This could lead to the use of suboptimal statin intensity for patients with high and very high CVD risk. The gap of knowledge regarding statin intensities has been shown in previous work from Suadi Arabia, where the questions about statin monitoring and the high-intensity statin group had the lowest percentage of correct answers with 9.1% and 28.6%, respectively [10]. In Jordan, more than half of the physicians were able to define the moderate- and high-intensity statin groups and gave correct examples for each of them [11].

Alarmingly, approximately three-fifths of respondents in the present study were unaware that DM patients aged 40–75 with LDL-c 70-189mg/dl should receive at least moderate-intensity statin. Their answers were either they do not need a statin or should receive a low-intensity dose. Rababa’h et al. (2020) found that only 45.4% of participating Jordanian physicians recommended the right therapeutic statin plan for diabetic patients aged 40–75 years old [11]. Although several studies worldwide have reported the underutilization of statins among patients with DM aged 40–75 years old, in our study, we confirm the lack of knowledge among Yemeni physicians and pharmacists regarding statin use in DM patients as a potential reason for the underutilization of statins among patients for primary prevention in Yemen, particularly diabetic patients aged 40–75 years old.

Knowledge gaps were found among pharmacists and physicians with regard to the time of statin therapy intake and the awareness about the statin that does not need renal adjustment in CKD. The knowledge gaps were significantly higher among physicians compared to pharmacists. A possible explanation for this is that pharmacists are exposed more to information regarding the time of drug intake and dose adjustments in CKD patients, and one of their roles is to counsel the patients and/or physicians about such information. Just over a fifth of respondents knew that atorvastatin and rosuvastatin could be administered at any time of the day. Many patients prefer to take the medication in the morning, and forcing them to take it at a specific time could contribute to their non-adherence to statin therapy. Therefore, healthcare providers’ knowledge that some statins can be taken at any time of the day is fundamental, and it can help match the patients’ preferences about the time of statin intake.

Similarly, prescribing a statin for patients who have CKD is common, and physicians and pharmacists should be aware of statins that do not need a dose adjustment and are preferable to use in such cases as atorvastatin. Using the statins such as rosuvastatin in CKD patients requires dose limitations. Thus, physicians’ and pharmacists’ awareness of the safest agents and the recommended doses in CKD patients is vital to minimize statin-related side effects.

Pharmacists and physicians had a low level of knowledge regarding statin-drug interactions, and the correct response rate is significantly lower among physicians. This gap of knowledge has been reflected in Yemeni clinical practice, where 18% of patients on a statin had a clinically significant statin-drug interaction [29]. These findings are similar to those of an American study that reported knowledge-practice gaps with regard to dyslipidemia management and underlined the necessity of education [30]. CME, such as workshops or/and lectures, may be beneficial in these situations [31, 32]. More practically and less time-consuming, it is best to integrate an alert system for clinically significant DDIs in the hospital or pharmacy information system. Previous studies have demonstrated that integrating guideline recommendations or alert systems into the IT system improves treatment and decreases the DDI rate [33–35]. However, Yemen is still far behind in integrating technology into the healthcare system, and most of the prescriptions are still handwritten. Therefore, it appears more suitable, at least at the moment, for most Yemeni healthcare providers to screen for potential SDIs using reliable DDIs software.

Healthcare providers’ knowledge about contraindications for drugs is important, and when we talk about statins that are utilized by millions of people, it becomes more prominent as the consequences would be bigger. In the present study, suboptimal awareness about statin contraindications was observed among the respondents. The highest knowledge gap was for the question related to the use of statins among breastfeeding women, where a significant percentage of respondents thought it safe to use. Similarly, around two-fifths of participants did know that statins are contraindicated in pregnancy. Currently, statins are contraindicated in pregnancy and lactation by the FDA and the manufacturers. However, changes in such recommendations could occur in the near future. To illustrate, contrary to animal studies and early uncontrolled case series reports that highlighted the potential teratogenic effect of statins [36–38], a recent observational study reported no elevated risk of congenital malformations related to statin exposure in pregnancy when compared to control groups [39]. However, the same study found an increased risk of low birth weight and preterm birth with statin use. Furthermore, the impact of statins on three key pregnancy-related outcomes (spontaneous abortion, stillbirth, and preterm delivery) have been assessed in a recent systematic review and meta-analysis [40]. The authors concluded that statin therapy appears to be safe as it was not associated with preterm delivery or stillbirth but associated with a significant increase in the risk for spontaneous abortion. The authors also emphasized the need for future studies to determine whether statin therapy could be beneficial in a subset of pregnant women, for whom the potential benefits of statin use outweigh the risk [40]. On July 20, 2021, the FDA requested that the prescription material for the whole class of statin drugs to be revised regarding usage during pregnancy [41]. These modifications include the removal of the contraindication label in all pregnant women. According to the FDA, such actions are expected to allow health care professionals to make individual judgments about benefits and risks, particularly for individuals at very high risk of heart attack or stroke. This includes individuals with homozygous familial hypercholesterolemia and those who have previously suffered from ASCVD.

### Association between knowledge and sociodemographic data

Younger participants, having <6 years of experience, working in community pharmacies, GPs, and pharmacists with diploma degrees were associated with a low level of statins’ clinical knowledge. These findings are in line with results reported by other studies. For example, younger Kuwaiti physicians were less knowledgeable about lipid guidelines and consequently used them less frequently [28]. Similarly, a Jordanian study reported that older age and higher rank of physicians were associated with better knowledge about cholesterol guidelines and that gender and hospital type were not significantly associated with knowledge [11]. Furthermore, in a study from Singapore, cardiologists were more familiar with the 2013 ACC/AHA recommendations than nephrologists, endocrinologists, or general practitioners [16]. In addition, a study from Suadi Arabia found that specialty significantly affected the physicians’ and clinical pharmacists’ knowledge about dyslipidemia guidelines [10]. Moreover, healthcare providers with postgraduate qualifications had better knowledge and practice regarding statin use among diabetic patients [42].

On the other hand, other studies in literature reported contrary findings. In this light, more knowledge gaps were identified among USA clinical pharmacists with more experience and who specialized in internal medicine [15]. Similarly, Zaitoun et al. reported a higher knowledge level among the female gender than males [10]. On top of that, a recent study found no significant difference in the appropriate utilization of statin between patients managed by general internists versus groups managed in collaboration with a cardiologist [43].

### Knowledge about monitoring parameters

There were knowledge gaps among the respondents regarding monitoring parameters, and they are higher among pharmacists. In this light, a higher percentage of pharmacists lacked the knowledge about the recommended duration for doing lipid profiles for follow-up (4–12 weeks) and routine measurements (3–12 months). Moreover, a significantly higher percentage of physicians than pharmacists choose ALT monitoring at baseline. This was expected as monitoring statins’ efficacy and safety is considered part of physicians’ duties in Yemen. Nevertheless, more than two-fifths of physicians were unaware that a lipid profile should be performed 4–12 weeks after statin initiation or dose change to assess the response and adherence. The knowledge gaps regarding monitoring parameters corroborate previous findings of suboptimal awareness and/or inappropriate practices toward statins’ monitoring in a clinical setting among healthcare providers in different countries such as the USA [14], Jordan [11], KSA [10], Malaysia [26].

For statins’ efficacy monitoring, there is some kind of consensus between the different guidelines regarding lipid monitoring at baseline, follow-up (4–12 weeks after initiating a statin therapy or dose change), and routine measurement (3 months to one year). However, there are some differences in recommendations regarding safety monitoring. For example, the 2018 ACC/AHA guideline has no clear recommendation regarding routine HbA1c or glucose monitoring [5]. On the other hand, the 2019 ESC/EAS guideline recommends that regular HbA1c or glucose testing be considered in individuals with high-dose statin therapy or at high risk of developing diabetes [6]. Another key difference regarding CK and ALT monitoring at baseline and follow-up. The 2018 ACC/AHA guideline has an expert opinion to do ALT at baseline but does not recommend it at follow-up unless clinically indicated. For CK monitoring, it is not recommended at baseline nor at follow-up unless clinically indicated (high risk for myopathy or syptoms developed) [5]. On the other hand, the 2019 ESC/EAS guideline recommends ALT and CK monitoring at baseline and follow-up. Regarding the routine measurement of ALT and CK, both guidelines do not recommend that [6].

### Barriers to guideline implementation in Yemeni clinical practice

The barriers reported by physicians and pharmacists that hinder the implementation of the 2018 ACC/AHA cholesterol guideline were analyzed. The most common barriers were no audit on adherence to the guidelines in the workplace, insufficient resources to implement and follow up the guideline’s recommendations adequately, and patients’ financial status. In contrast, the patient’s inability to afford medications was reported by a minority of healthcare providers in the USA [14]. This difference is expected due to the fact that the healthcare system in Yemen is semi-collapsed, with no government health insurance. Additionally, a large percentage of the population is in need of humanitarian assistance and cannot afford private health insurance or costly medications on a regular basis [44]. Providing patients in need with chronic medication such as statins can improve the utilization of statins for those with a high risk for CVD. In fact, there is currently the Yemen Medicine Bank. It is a non-profit organization that aims to provide urgent and necessary health and pharmaceutical services that contribute to reducing disease for the poor and most vulnerable groups. One of its programs is to provide the needy, the poorest in society, and those with chronic diseases with free medicines needed for them every month. However, the program coverage is still shallow, and more government and private sector efforts are needed to mitigate the patients’ financial status barrier [45].

In our analysis, a minority of healthcare providers reported disagreement with guidelines, rolling out the negative attitude as a major player in guideline implementation in Yemen. Attitudes-based barriers have been traditionally reported among healthcare providers with different degrees of prevalence among studies. For example, disagreement with the dyslipidemia guideline was reported by 11.3% of providers in a USA-based study [14]. However, disagreement among the USA clinical pharmacists was higher (23.5%) [15]. Similarly, attitude-related obstacles have been reported among Kuwaiti physicians [28].

Several studies have reported a lack of knowledge as a barrier to dyslipidemia guideline implementation. This was affirmed in our study in which a high proportion of respondents perceived a lack of familiarity with guidelines as a barrier to implementation. These findings highlight the need for educational interventions in the form of CME to improve the healthcare provider’s knowledge of the up-to-date guideline recommendations. Other methods have been proposed previously to overcome these obstacles, such as integrating guideline’s key recommendations into the workplace system, developing local guidelines, and presenting key recommendations in various formats, including one- or two-page summaries or algorithms [28].

While this phase accurately captured participants’ clinical knowledge of statin therapy and monitoring parameters, some limitations existed. To begin, data was solely collected in Sana’a. This may limit the generalization of the findings. Second, because convenience sampling was used, this could lead to selection bias. Despite these limitations, the study includes an adequate sample size. Also, this is the first study to comprehensively assess clinical knowledge about statin medication, and some of the questions in the knowledge section were constructed for the first time in this study based on recent guideline guidelines. It is also the first study from Yemen to give helpful information on the barriers to cholesterol management guideline implementation.

## Conclusion

Physicians and pharmacists had suboptimal clinical knowledge regarding statin therapy, dose intensities, drug-drug interaction, contraindications, and monitoring parameters. The most common barriers to implementing cholesterol guidelines were no audit on adherence to the guidelines in the workplace, insufficient resources to implement and follow up the guideline’s recommendations adequately, lack of knowledge about guidelines, and patients’ financial status. Therefore, physicians’ and pharmacists’ educational interventions regarding evidence-based medicine such as statins are recommended.

## Supporting information

S1 File Study questionnaire. (DOCX)

S2 File Study dataset. (SAV)
