# Supplementary material for: Physicians and pharmacists’ clinical knowledge of statin therapy and monitoring parameters, and the barriers to guideline implementation in clinical practice
Source: PLoS One. 2023 Jan 20;18(1):e0280432. doi: 10.1371/journal.pone.0280432 (PMC9858478; doi:10.1371/journal.pone.0280432)
Supplement: S1 File — (DOCX) [file pone.0280432.s001.docx]

Physicians and pharmacists clinical knowledge regarding statin therapy: Clinical indications, DDI, contraindication, dose intensity utilization, and monitoring parameters

| **In this questionnaire, we would like to know your knowledge about statin therapy and its ‎monitoring parameters.** | | | |
| --- | --- | --- | --- |
| **SECTION A: Demographic data** | | | |
| 1. **Gender** | Male Female | | |
| 1. **Age** | ………………. | | |
| 1. **Degree** | **For physicians** | **For pharmacists** | |
|  | Consultant ‎  Specialist ‎  Resident doctor ‎  General practitioner | Pharm.D  Bachelor degree (B.Pharm)  Diploma | |
| 1. **Current Working place** | **For physicians** | **For pharmacists** | |
|  | Private Hospital  Govermental hospital  Private Clinic  Others (please specify): | Community pharmacy  Private Hospital pharmacy  Govermental hospital pharmacy  Others (please specify): | |
| 1. **How many years have you been in practice?** | | | …………………. |
| 1. **Did you read any guidelines on cholesterol management during the last three years?** | | | 􀁆 Yes 􀁆 No |

| Section B: Knowledge about Statin therapy | | |
| --- | --- | --- |
| 1. It is recommended to initiate statin therapy without ASCVD risk assessment in all the following patients EXCEPT: | | |
| 􀁆 Patients with acute coronary syndrome | | |
| 􀁆 Diabetic patients aged 45 years old with LDL-C levels of 70mg/dl or more. | | |
| 􀁆 Patients with a history of ischemic stroke | | |
| 􀁆 Obese and smoker patient aged 42 years old | | |
| 􀁆 I do not know/ Not sure | | |
| 2. What percent of LDL-C reduction would you expect from low, moderate and high-intensity statin daily therapy? | | |
| 􀁆 <10 % for low, 10-<30 % for moderate, and ≥ 30 % for high-intensity statin therapy. | | |
| 􀁆 <20 % for low, 20-<40 % for moderate, and ≥40 % for high-intensity statin therapy. | | |
| 􀁆 < 30 % for low, 30- <50 % for moderate, and ≥50 % for high-intensity statin therapy. | | |
| 􀁆 I do not know‏/‏‎ Not sure . | | |
| 3. Which of the following daily doses of statin therapy is considered a high-intensity statin: | | |
| 􀁆Atorvastatin 20mg | 􀁆Rosuvastatin 20mg | 􀁆Pravastatin 80 mg |
| 􀁆Simvastatin 40mg | 􀁆Fluvastatin 40 mg | 􀁆I don’t know‏/‏‎ Not sure |
| 4. Which of the following daily doses of statin therapy is considered a moderate-intensity statin: | | |
| 􀁆Rosuvastatin 10 mg | 􀁆Pravastatin 20 mg | 􀁆Simvastatin 10mg |
| 􀁆Fluvastatin 40 mg/day | 􀁆I don’t know/ Not sure |  |
| 5. Which of the following statin-intensity is recommended for adult patients with clinical ASCVD such as myocardial infarction and angina: | | |
| 􀁆 Low-intensity statin 􀁆Moderate intensity statin 􀁆 High-intensity statin 􀁆 I do not know | | |
| 6. Which of the following statin-intensity is recommended for adult patients with severe primary hypercholesterolemia (LDL-C level ≥190 mg/dL [≥4.9 mmol/L]): | | |
| 􀁆 Low-intensity statin 􀁆Moderate intensity statin 􀁆 High-intensity statin 􀁆 I do not know | | |
| 7. Which of the following statin-intensity is recommended for a 40-year patients with only diabetes mellitus type 2 with with LDL-C level of 80 mg/dL: | | |
| 􀁆 No need for statin therapy 􀁆 Low-intensity statin 􀁆Moderate intensity statin 􀁆 I do not know | | |

| 8. Which of the following statin medications can be taken by patients at any time of the day? | | |
| --- | --- | --- |
| 􀁆No one, all statins must be administered at night | |  |
| 􀁆Pravastatin only | 􀁆Simvastatin only | 􀁆Simvastatin and Pravastatin |
| 􀁆Atorvastatin and Rosuvastatin | 􀁆I don’t know |  |

| 9. Which of the following statin medications does not need dose adjustment in chronic kidney disease: | | | | | |
| --- | --- | --- | --- | --- | --- |
| 􀁆 Simvastatin | | 􀁆 Atorvastatin | | 􀁆 Rosuvastatin | 􀁆 I do not know |
| 10. Which of the following statin medications is associated with clinically significant drug-drug interaction when used in combination with *amlodipine*: | | | | | |
| 􀁆 Simvastatin 􀁆 Atorvastatin 􀁆 Rosuvastatin 􀁆 I do not know | | | | | |
| 11. Which of the following statin medications is preferred to use for patients on warfarin to avoid drug-drug interactions: | | | | | |
| 􀁆 Simvastatin 􀁆 Atorvastatin 􀁆 Rosuvastatin 􀁆 I do not know | | | | | |
| 12. Which of the following statin medications is the safest to use in patients taking clarithromycin: | | | | | |
| 􀁆 Simvastatin 􀁆 Atorvastatin 􀁆 Rosuvastatin 􀁆 I do not know | | | | | |
| 13. Statin therapy is contraindicated in pregnancy: | | | | | |
| 􀁆 Yes | 􀁆 No | | 􀁆 I do not know/not sure | | |
| 14. Statin therapy can be used safely during breastfeeding | | | | | |
| 􀁆 Yes | 􀁆 No | | 􀁆 I do not know/not sure | | |
| 15. Statin therapy is contraindicated in active liver disease: | | | | | |
| 􀁆 Yes | 􀁆 No | | 􀁆 I do not know/not sure | | |

| **C: Knowledge about monitoring parameters** | |
| --- | --- |
| **1. Which of the following lab tests is/are recommended for the patients at baseline before initiating statin therapy?** | |
| ***PLEASE SELECT ALL THAT APPLY*** | |
| 􀁆 Lipid levels (Total cholesterol, HDL, LDL, TG) | 􀁆 HbA1c |
| 􀁆 Fasting glucose | 􀁆 Liver enzymes (ALT/AST) |
| 􀁆 Creatinine kinase (CK) | 􀁆 I do not know/ not sure |
| **2. It is recommended to assess statin efficacy by measuring LDL-C ……………… after statin ‎therapy initiation or dose change:‎** | |
| 􀁆 2-3 weeks 􀁆 1-3 months 􀁆 4-6 months 􀁆6-12 months 􀁆 I am not sure | |
| **3. Once the patient achieved the target lipid level, it is recommended to do a lipid profile every:** | |
| 􀁆 2-3 weeks 􀁆 1-3 months 􀁆 3-12 months 􀁆 I am not sure | |
| **Section D: Barriers to guideline adherence** | |
| **What are the possible reasons that you may not be able to follow and implement the guidelines on the treatment of blood cholesterol in your practice? Below we have listed many possible reasons.**  ***PLEASE CHOOSE ALL THAT APPLY TO YOU:***  *􀁆* Not very familiar with the guideline latest recommendations for statin therapy  􀁆 Workload and lack of time prevent the application of guideline recommendations  􀁆 Patients’ financial status does not allow to implement the guideline recommendation ‎  􀁆 Adherence to the international guidelines will not make a difference in the clinical outcomes of Yemeni patients  􀁆 I do not agree with some of the recommendations regarding statin therapy  􀁆 The cholesterol management guideline is too complex and difficult to use  􀁆 The cholesterol management guideline is too long  􀁆 The cholesterol management guideline changes too often  􀁆 The cholesterol management guideline is too rigid to apply to individual patients  􀁆 There is no follow-up or audit on guideline adherence in the workplace  􀁆 There are not enough resources to adequately implement and follow up on the ‎guideline’s recommendations  􀁆 The patients will not be compliant with statins due to side effects (e.g., myalgia)‎  􀁆 Others (Please specify):‎ | |
